# Decoding Motor Imagery through Common Spatial Pattern Filters at the EEG Source Space

**DOI:** 10.1155/2018/7957408

**Published:** 2018-08-01

**Authors:** Ioannis Xygonakis, Alkinoos Athanasiou, Niki Pandria, Dimitris Kugiumtzis, Panagiotis D. Bamidis

**Affiliations:** ^1^Biomedical Electronics Robotics and Devices (BERD) Group, Lab of Medical Physics, School of Medicine, Faculty of Health Sciences, Aristotle University of Thessaloniki (AUTH), 54124 Thessaloniki, Greece; ^2^Department of Electrical and Computer Engineering, Faculty of Engineering, Aristotle University of Thessaloniki (AUTH), 54124 Thessaloniki, Greece

## Abstract

Brain-Computer Interface (BCI) is a rapidly developing technology that aims to support individuals suffering from various disabilities and, ultimately, improve everyday quality of life. Sensorimotor rhythm-based BCIs have demonstrated remarkable results in controlling virtual or physical external devices but they still face a number of challenges and limitations. Main challenges include multiple degrees-of-freedom control, accuracy, and robustness. In this work, we develop a multiclass BCI decoding algorithm that uses electroencephalography (EEG) source imaging, a technique that maps scalp potentials to cortical activations, to compensate for low spatial resolution of EEG. Spatial features were extracted using Common Spatial Pattern (CSP) filters in the cortical source space from a number of selected Regions of Interest (ROIs). Classification was performed through an ensemble model, based on individual ROI classification models. The evaluation was performed on the BCI Competition IV dataset 2a, which features 4 motor imagery classes from 9 participants. Our results revealed a mean accuracy increase of 5.6% with respect to the conventional application method of CSP on sensors. Neuroanatomical constraints and prior neurophysiological knowledge play an important role in developing source space-based BCI algorithms. Feature selection and classifier characteristics of our implementation will be explored to raise performance to current state-of-the-art.

## 1. Introduction

Brain-Computer Interface (BCI) is emerging as a promising rehabilitation technology, that aims to establish a connection between brain activity and external devices. Recent advances in invasive BCIs have demonstrated the feasibility of performing complex motor tasks using brain signals by people with disability such as severe spinal cord injury and quadriplegia [1]. As invasive BCIs use intracranial electrodes to measure electrical activity of the cerebral cortex, either implanted or directly lying on the cortical surface such as electrocorticography (ECoG), their usage is limited due to ethical, medical, and physiological issues [2]. These limitations are not present with noninvasive BCIs, and the most widely used noninvasive modality, electroencephalography (EEG), uses electrodes over the scalp to measure inferred cerebral cortical activity.

A variety of brain signal types and features have been used to decode user intent in noninvasive EEG-based BCIs, such as visual evoked potential (VEP), P300 response, slow cortical potentials (SCP), and sensorimotor rhythm (SMR), to name but a few [3]. SMR or mu (*μ*) rhythm, typically measured at the alpha band of 8–13 Hz over the scalp area overlying the sensorimotor cortex, can be modulated during motor execution or motor imagery (MI) tasks, and the BCIs decoding this type of signal are referred to as SMR-BCIs. Motor imagery displays similar patterns of brain activation and communication to motor execution [4, 5] while research and development in the domain of SMR-BCIs has brought some remarkable applications ranging from the accurate control of a cursor in 2-D space [6], control of a quad-copter in 3D space [7], control a robotic arm for reach and grasp tasks [8], and control of a wheelchair [9] proving the potential of this technology.

Nonetheless, noninvasive BCIs also feature a number of limitations with regards to reliability, speed, and accuracy and have many challenges to overcome to meet both research and casual everyday use needs. Key features for the success of SMR-BCIs involve the classification accuracy, performance robustness, and asynchronous and intuitive control that requires the decoding of multiple motor imagery tasks. Control of an external complex device with multiple degrees of freedom, such as a robotic arm or an artificial limb, can be better achieved by utilizing motor imagery classes that are related to the intended end effector movement [10, 11], making control more intuitive and thus requiring less time for training.

Moreover, intrinsic drawbacks of EEG include low signal-to-noise ratio (SNR), low spatial resolution, and imprecise and indirect measuring of brain activity mainly attributed to the volume conduction effect. This effect describes the spread of the brain's electrical field while it is transmitted from the source space through the cerebrospinal fluid, skull, and scalp to reach the scalp surface where the electrodes lay, known as the sensor space [12]. To reduce the volume conduction effect and study the brain activity on the cortex, source imaging techniques are commonly used that map the scalp potentials measured by EEG sensors to cortical activations on the cortical mantle [13, 14]. Low SNR led the researches to search for spatial filters that extract the EEG components that reflect user intention. In this context, Common Spatial Pattern (CSP) method was proposed to extract spatial features of event related de/synchronization during motor imagery [15]. CSP filters are spatial filters designed to maximize the power difference on their outputs given different EEG classes [15]. CSP filters are considered as an effective way to discriminate classes and are one of the most popular feature extraction methods in the BCI field [16], which also have multiple extensions [17–20]. Remarkable classification results have already been reported by studies that implemented the CSP algorithm or its variants [21, 22].

In the current study, we describe the development of a BCI algorithm, aiming to decode multiple (4) MI tasks. In order to overcome the issues associated with low spatial resolution, we use source imaging and extract features in the cortical source space from selected Regions of Interest (ROIs), using Common Spatial Pattern filters. Finally, the classification is performed with an ensemble classification model that synergistically uses the classification models of selected ROIs, in order to increase classification accuracy.

## 2. Materials and Methods

### 2.1. BCI Competition Dataset

The BCI Competition IV 2a dataset was used to develop and test the BCI decoding algorithm. The dataset contains recordings from 9 healthy subjects that perform 4 motor imagery tasks, right arm, left arm, feet, and tongue [23]. The data of a subject consist of 2 sessions, one intended for training and the other for evaluation. Each session is comprised of 72 trials for each MI task, 288 trials in total, recorded with 22 EEG channels and 3 monopolar electrooculogram (EOG) channels (with left mastoid serving as reference). In our study, only data from the training session are used.

**Figure 1:**
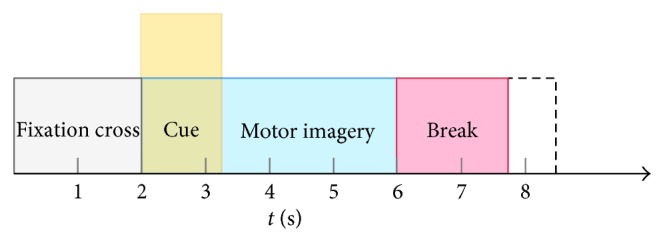
Diagram of a trial and timings during a session of the BCI Competition IV 2a dataset.

At the beginning of each trial (*t* = 0 s), a white cross on black background appeared, and after 2 s, an arrow oriented right, left up, or down informed the subject to perform the corresponding MI task (Figure 1). The arrow appeared for 1.25 s, and the subject was asked to keep on performing the MI task until the white cross disappeared (*t* = 6 s).

### 2.2. Signal Preprocessing

Signal analysis was performed solely on the EEG electrodes, and the EOG channels were excluded. Average reference was used, and the data were band-pass filtered at 7–15 Hz using a zero-phase FIR filter in order to capture the event related desynchronization and synchronization (ERD/ERS) activity [24]. Subsequently, data were down-sampled at 100 Hz and epoched for 500 msec after the visual cue with epoch duration of 3000 msec. Data were visually inspected for bad channels but none was excluded. All preprocessing was performed using a custom Fieldtrip script [25].

### 2.3. Inverse Problem Solution

EEG source imaging was deployed to mitigate low spatial resolution and low SNR caused by volume conduction. EEG source imaging maps sensor activity to brain neural current distribution at fixed positions over the cortex. The source activity is defined in terms of current dipoles, at a grid of vertices on the MNI cortical surface template, that model electrical activity of neuronal groups firing synchronously [26, 27]. The estimation of the sources from the EEG recordings constitutes the solution for the inverse problem, while the forward problem is described by the following equation (assuming zero noise) [27]:

(1) $$\begin{array}{c} M=GD, \end{array}$$

where *M* is the *N*_c_ × *T* matrix of the EEG data, *G* is the lead-field matrix (also referred as gain matrix) that maps the source data to sensor data (*N*_c_ × *N*_d_), and *D* is the dipole current density (*N*_d_ × *T*). *N*_d_ is the number of current dipoles, *N*_c_ is the number of EEG channels, and *T* is the number of measurements. Solving the forward problem consists of computing the lead-field matrix, referred to as head or forward model, that models how current flows from the sources through different head compartments (scalp, skull, and cortex) to the scalp surface.

The Montreal Neurological Institute (MNI) Colin 27 MRI generic template [28] was used as the default subject anatomy to compute a three-compartment (scalp, skull, and cortex) head model with symmetric boundary element method (BEM) using OpenMEEG [29]. Default Brainstorm Colin 27 cortex was down-sampled using iso2mesh [30] to 5023 vertices, and the relative conductivity values of Scalp/Skull/Brain was assumed to be 1 : 1/15 : 1, with *σ*_brain_=*σ*_scalp_=0.33  S/m and *σ*_skull_=0.0042  S/m [31]. All 5023 dipoles are assumed constrained to the cortical surface with an orientation perpendicular to the surface, based on the assumption that EEG primary signal sources are local groups of pyramidal neurons firing synchronously, located on the cortex and arranged perpendicular to its surface [26, 31].

Given the lead-field matrix, the inverse EEG problem consists of finding the dipole current density D in (1). This is a highly underdetermined problem since the number of dipoles (sources) is at the order of thousands and the number of EEG channels is at most at the order of hundreds, which in practice means that different current distributions (brain activity) can lead to exact EEG sensor values. Among different methods for solving the inverse problem, here it was solved with the weighted minimum norm estimate (wMNE) method using the Brainstorm toolbox [32–34]. Sensor noise covariance matrix, required for the computation of the solution, was calculated on the resting state period at the start of the session.

### 2.4. Regions of Interest

**Figure 2:**
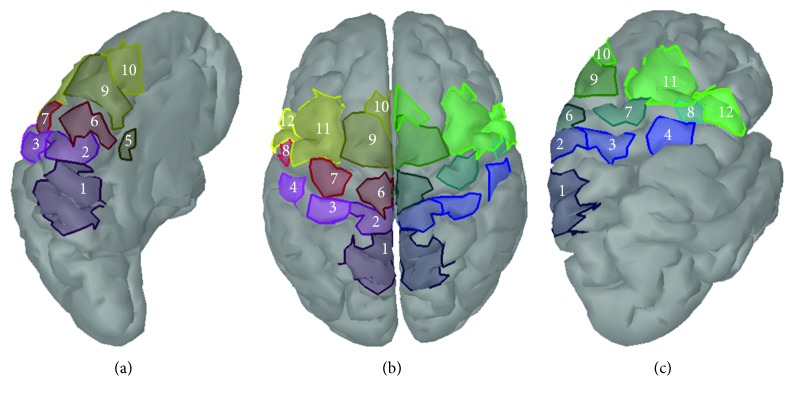
Regions of Interest (ROIs) at the cortical level: (a) midline surface, left hemisphere, (b) top view, both hemispheres, and (c) lateral view, right hemisphere. 1: SAC, 2: S1F, 3: S1H, 4: S2, 5: CMA, 6: M1F, 7: M1H, 8: M1L, 9: SMA, 10: pSMA, 11: PMd, 12: PMv [36].

Cortical Regions of Interest (ROIs) were defined on the sensorimotor cortex to reduce the dimension of the source data derived from the inverse problem solution, having anatomical constraints and aiming at extracting valuable information related to MI tasks [11, 35]. 24 ROIs were defined based on neuroanatomical landmarks and Broadman areas and are depicted in Figure 2. Defined ROIs include bilaterally presupplementary motor area (pSMA), supplementary motor area (SMA), cingulate motor area (CMA), dorsal premotor cortex (PMd) and ventral premotor cortex (Pmv), primary foot motor area (M1F), primary hand motor area (M1H) and primary lip motor area (M1L), primary foot somatosensory area (S1F), primary hand somatosensory area (S1H), secondary somatosensory area (S2), and somatosensory association cortex (SAC). During ROI analysis, source times-series that lay only on the defined ROIs on the mantle are analyzed, excluding from analysis all the other sources.

### 2.5. Feature Extraction

Feature extraction was performed at the source level, on ROIs data in particular. Common Spatial Pattern (CSP) filters are one of the most used feature extraction methods in BCI domain [16]. Assuming data of two classes, for example, the motor imagery of right and left, CSP algorithm calculates spatial filters that maximize the ratio of variance of data stemming from the two classes. Consequently, the extracted signals are optimally discriminating two different EEG classes while they are revealing spatial patterns of different classes [15, 17]. The spatially filtered signal *S* of an EEG trial is given by

(2) $$\begin{array}{c} S=WM, \end{array}$$

where *M* is a *N*_c_ × *T* matrix representing the EEG measurement of data for the given trial and *W* is *L* × *N*_c_ matrix referred as CSP projection, whose rows are the spatial filters designed to output signals whose ratio of variances are maximally discriminating input data of two different classes.

Original CSP algorithm has been developed for two class problems, though there exist multiclass extensions [17, 37]. Since the classification problem of this work is multiclass, a multiclass extension of CSP was deployed using the One-vs-Rest scheme, with *L*=8 filters, the last and the first eigenvectors of each class were selected [15]. CSP filters were calculated during training phase, on the mean covariance matrices of the data conditioned to the four classes.

In this work, CSP filters were applied to the source data, and they were calculated on every ROI current dipole time-series. Assuming *D*_q_ the current dipole times-series of ROI_q_, that resulted from the solution of the inverse problem (1), and *W*_q_ the CSP filter computed on the data of ROI_q_, the output of the ROI-CSP filters is

(3) $$\begin{array}{c} Z=W_{q}D_{q}. \end{array}$$

The feature vector of ROI_q_, *v*_ROI_q__ ∈ *ℝ*^*L*^ is extracted from CSP filters output, and each of its components, *v*_p_, *p* = 1,…, *L*, is given as

(4) $$\begin{array}{c} v_{p}=\log\left(\frac{\mathrm{var}\left(Z_{p}\right)}{\sum_{i=1}^{L}\mathrm{var}\left(Z_{i}\right)}\right)\in R,p\in \left[1,\;\;L\right], \end{array}$$

where *Z*_p_ is the *p* row of the matrix *Z*, that is, the output signal of the *p*th CSP filter output. Repeating this procedure for each selected ROI results to *Q* feature vectors of *L* = 8 elements, where Q is the number of selected ROIs.

### 2.6. Classification

**Figure 3:**
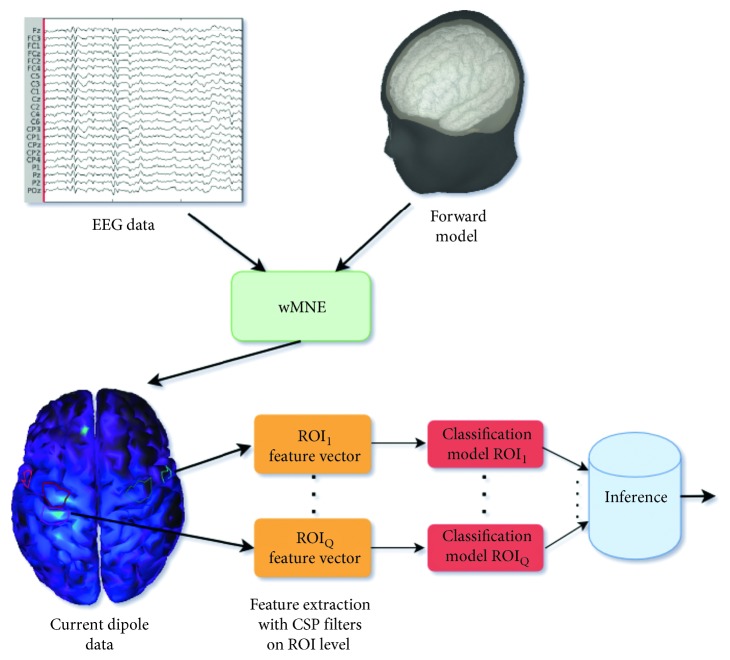
Outline of the implemented decoding algorithm. EEG sensor time series are transformed to current dipole time series. Data from the Regions of Interest (ROIs) are spatially filtered by ROI-CSP filters, to extract features to be classified by independent ROI classification models. Predicted class is the most voted class of the ROI classification models. On the classification model scheme, the predicted class is the outcome of an inference mechanism (majority vote). The inference mechanism takes as input the predicted class from the individual ROI classification models.

An ensemble classification model was used for the prediction of the MI task [38], illustrated in Figure 3. It is supported that an ensemble method using multiple independent classification models can increase the classification performance [39, 40]. An independent classification model was built for each of the selected ROIs, and the final classification outcome was selected by an inference (fusion) mechanism. *K*-nearest neighbors (kNN), Naive Bayes, Decision Tree, and Linear Discriminant Analysis (LDA) classifier were tested with LDA having superior performance as it is demonstrated in the Results section. The ROI classification model was based on LDA, and the inference mechanism was the majority vote of the selected ROI classification models. The selected ROIs were the Q most accurate ROIs according to a selection procedure that is presented in the next section.

### 2.7. ROI Selection

The defined ROIs extend all over the motor cortex, while the cortical activity related to the performed motor imagery tasks is derived only from a subset of the defined ROIs. ROIs were selected based on their classification model accuracy. In order to select the most accurate ROIs, 10-fold cross-validation using the LDA classifier was performed on ROI level, and this was repeated 10 times to ensure more robust results (in every run, CSP filters are calculated on different data). The *Q* = 8 most accurate ROIs were selected. The number is based on parametric analysis results of the inference mechanism accuracy. Parametric analysis was run for different subjects, and the number of selected ROIs was set to *Q* = 8.

The performance of the classification scheme on the source space was further compared to the performance on the sensor space using the same setting (10-fold cross-validation of the LDA classification repeated 10 times). For the sensor space, the CSP filters are computed on the preprocessed EEG data. Moreover, to better assess the developed method, performance in terms of Cohen's kappa statistic, a useful metric for multiclass prediction problems, was compared to the winner of BCI Competition IV of dataset 2a [23, 33]. The winner of the competition deploys CSP on multiple frequency bands (FBCSP) as feature extraction method, Mutual Information-based Best Individual Feature (MIBIF) algorithm for feature selection, and Naïve Bayesian Parzen Window (NBPW) classifier [21, 41].

## 3. Results and Discussion

### 3.1. Classification Accuracy

**Figure 4:**
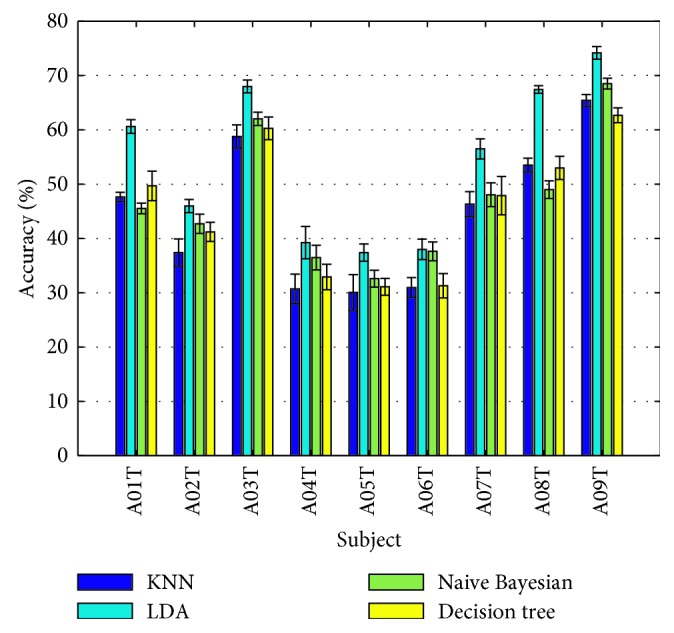
Classification accuracy of kNN, LDA, Naïve Bayesian, and Decision Tree classifiers across all subject data.

Four different classifiers were tested to select the classifier to make the predictions. LDA, kNN, Naive Bayesian, and Decision Tree were tested by performing 10-fold cross-validation, 10 times on all subjects. LDA had superior performance with the highest prediction accuracy among all subjects, with mean accuracy 54.1%. Naive Bayesian was second with 46.9%, followed by Decision Tree and kNN with 45.5% and 44.5%, respectively (Figure 4).

**Figure 5:**
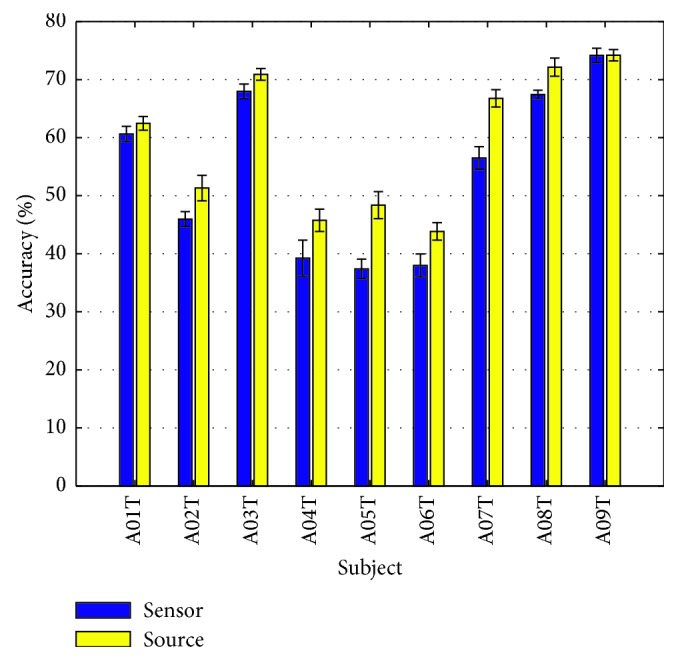
Classification accuracy of the developed source method and the equivalent traditional sensor approach, on the BCI Competition IV, 2a dataset.

The source method of classification achieved consistently higher accuracy rates across all subjects (43.7% to 74.5%), when compared to the sensor method (37.7% to 73.4%), as illustrated in Figure 5 and displayed in Table 1 below. Comparison of the developed method's performance to the winner of BCI Competition IV of dataset 2a in terms of Cohen's kappa statistic [42, 43] (multiclass prediction) is presented in Table 2.

Classification sensitivity and specificity, also referred to as true positive and true negative rate respectively, between the source and sensor method are demonstrated in Tables 3–6. Among the subjects, the source method has mean 11.1% higher true positive rate for the left arm, 5.2% higher for right arm, and 3.3% and 1.9% better rate for foot and tongue imagery, respectively. The mean differences of sensor to source true negative rate metric are low for all classes, −1.2%, 1.9%, 2.9%, and 3.6% for left, right arm, foot, and tongue imagery, respectively.

### 3.2. Selected ROIs

**Figure 6:**
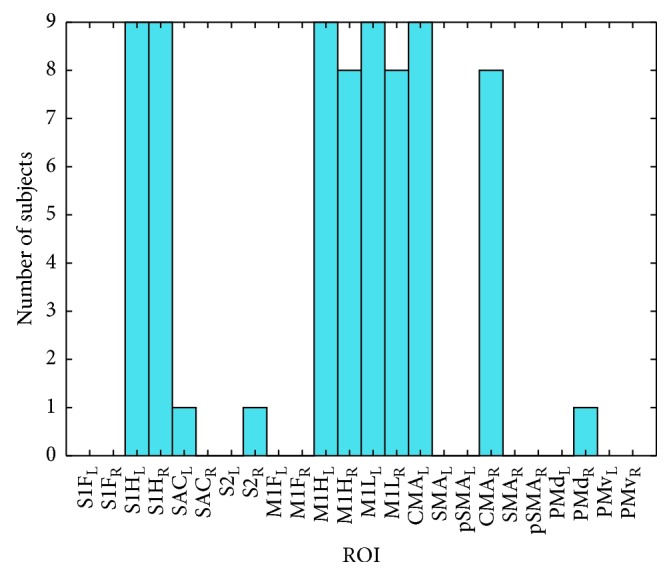
Histogram of the selected regions of interest (ROI), across all subjects.

**Figure 7:**
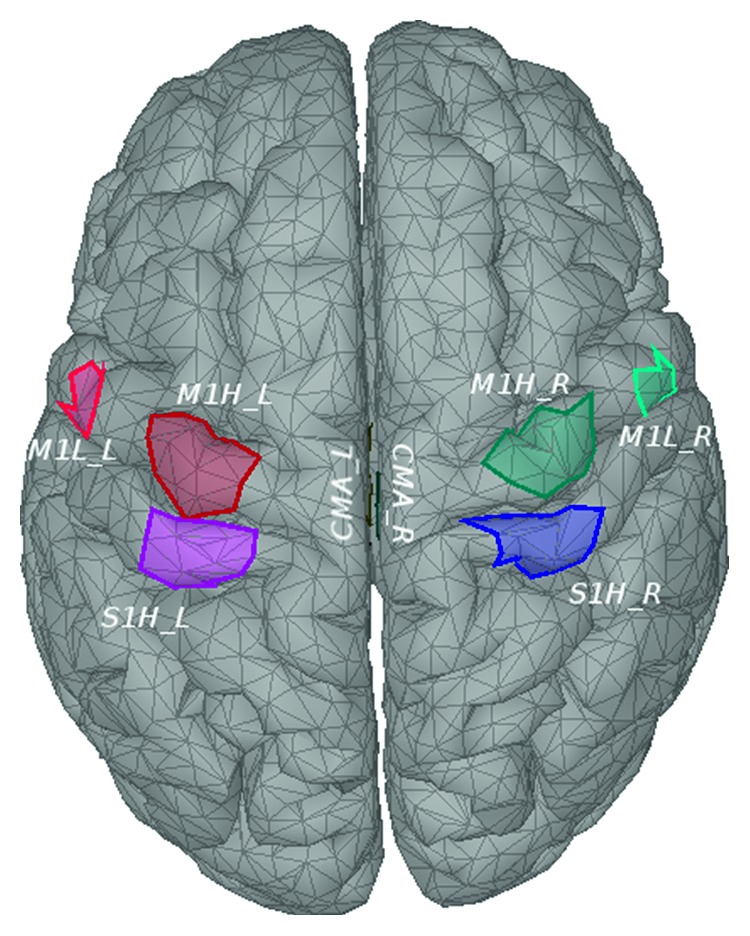
Positions of the most commonly selected ROIs among subjects (right and left M1L, M1H, S1H, and CMA), displayed on the cortical model.

The ROI selection procedure was performed for all the subjects, exhibiting interesting intersubject properties. As illustrated in Figure 6, the symmetrical left and right S1H, M1L, M1H, and CMA ROIs were the, *Q* = 8, most selected among all the subjects, with the left and right S1H, and left M1L, M1H, and CMA being selected for all 9 subjects. For the subjects A02T, A06T, A09T the pMd_R, SAC_L, and S2_R were selected instead of M1L_R, M1H_R, and CMA_R, respectively, with still 7 out of 8 selected ROIs being on the most frequent ones. Most frequently selected ROIs are illustrated on Figure 7 on the cortical mantle model.

### 3.3. Discussion

Noninvasive BCI systems emerge as a promising and safe solution for rehabilitation purposes in contrast with invasive BCIs that are associated to health risks and ethical issues [44], but their commercial use is still hindered by low performance and instability. Despite a number of already demonstrated SMR-BCI applications [8, 9, 45], noninvasive BCIs still suffer from low SNR. In our work, we investigate the use of source imaging and subsequent application of CSP on the source space to compensate for the head volume conduction by mapping scalp potentials to cortical activations [46]. There are several studies supporting that BCI algorithms based on source space features are superior to the sensor ones [11, 47], an observation that is confirmed in our study, comparing the classification results on the sensor and source space. Our BCI algorithm uses, in particular, sources belonging to select Regions of Interest (ROIs) on motor cortex for feature extraction and an ensemble classification model to take advantage of ROI data.

Despite the fact that our algorithm did not reach the accuracy levels of the wining method of the BCI Competition, during the ROI selection procedure, common ROIs emerged among all subjects. The emerged ROIs are anatomically and neurophysiologically related to the MI tasks, linking the method results with neurological data. Given that the motor tasks of the competition involved motor imagery of both arms, tongue, and feet, consistent selection of primary hand motor areas and primary lip motor area (cortical representations of hands and face on the primary motor cortex) seems very promising. Cingulate motor areas are also considered very important nodes of the sensorimotor network, having been demonstrated to drive the sensorimotor process [48, 49]. It is our conviction that selected ROIs, as produced by the developed algorithm, validate our results since there is a clear neuroanatomical and neurophysiological link between these ROIs and the Motor Imagery tasks performed in the dataset.

Our method appeared to improve mean accuracy by 5.6% and by 0.07 Cohen's kappa value among all subjects, with respect to sensor method. When our algorithm is compared with the winner of the BCI Competition (FBCSP), the mean accuracy is considerably lower by 0.19 Cohen's kappa value. The performance of our algorithm based on kappa value is considered moderate while that of the winning implementation is considered substantial [40]. We believe this difference is attributed to feature selection and classifier used by the winner. FBCSP generates CSP features in different frequency bands resulting to multiple features, while feature selection procedure is a vital component to detect the most discriminable features [41]. On the other hand, our source-based algorithm seems to increase the classification accuracy of subjects with the worst performance, namely the A04, A05, & A06, as it can be illustrated in Figure 5, outperforming sensor algorithm by a mean accuracy rate of 7.5%. Nevertheless, the trend identified cannot lead to safe conclusions yet, since we cannot infer statistical significance of the results, requiring further investigation on data with larger population of subjects. Moreover, ensemble classification was used, in an effort to increase classification accuracy, by synergistically deploying the independent ROI classification models. Majority vote of ROI classification models was used as final classification outcome, although a weighted vote taking into account the ROI-MI task relation could be considered in the future.

### 3.4. Limitations and Future Steps

In this study, a generic template three-compartment BEM head model was utilized to solve the forward problem. Forward problem solution induces an important error in the source estimation, as has been explored extensively in previous studies [50, 51]. Main forward problem error inducing factors are (a) the use of the MNI template MRI data rather than the subjects' individual neuroanatomy and (b) the absence of cerebrospinal fluid (CSF) in the forward modeling. Template anatomy was used for all subjects, missing important geometrical information for every subject, producing a lead-field matrix that transforms the EEG sensor data into a template cortical manifold different from the real one. CSF compartment has big influence on both signal topography and magnitude, resulting in strong signal attenuation for superficial sources on gyral crowns [52]. This effect is termed to the high increase of conductivity between the sensors and sources. In a future effort to address this problems, a 4- or 5-compartment head model including CSF and skull anisotropy will be used, modeled with finite elements [53].

There are two main shortcomings in the use of CSP method that were not dealt in this work. The first is that the CSP filters are prone to noise and overfitting, and the second is that the CSP performance is highly dependent on the input signal frequency band the individual subject BCI performance is dependable on individual frequency band used [20, 21]. There are many variants of conventional CSP algorithm designed to overcome the limitations, with popular variants being the RCSP that tackle noise and overfitting with regularization and the FBCSP that better capture the individual subject multiple frequency band filters feature selection, respectively, while a newly introduced method combines aforementioned methods [20, 21, 39].

Future work will focus on better CSP filters extraction and use feature selection and more sophisticated ensemble models, in an effort to increase the performance of the algorithm. Since the anatomy used for the forward model is common among all subjects and the selected ROIs are common among all subjects, we would like to check the potential of the algorithm in transfer learning between subjects. There is a study supporting that transfer learning between different subjects by means of source space can achieve higher average single-trial classification accuracy than with a conventional method [54]. Beyond the BCIC IV 2a dataset that is a common ground for the evaluation of methods decoding multiple MI, we aim to evaluate the improved method on dataset we compiled for the CSI: Brainwave project, containing EEG data of healthy or subjects with spinal cord injury performing multiple motor imagery mainly of the upper limbs [36, 55].

## 4. Conclusions

Source estimation and application of CSP filters at the source space constitute a promising solution to increasing classification accuracy of noninvasive BCIs. Our method has demonstrated capability in decoding multiple motor imagery tasks with better accuracy than the equivalent sensor method. While our implementation still is not superior to the state of the art of BCI algorithms, feature selection and classifier characteristics can improve performance. Neuroanatomical constraints and prior neurophysiological knowledge has been shown to play an important role in developing source space-based BCI algorithms. Our results indicate that the selected ROIs are common among all subjects, which worth further investigation probably in the context of transfer learning between different subjects.

## Figures and Tables

**Table 1:** 10 × 10-fold cross-validation performance in terms of mean classification accuracy (%) of the developed source method and the equivalent sensor method.

Subject	A01T	A02T	A03T	A04T	A05T	A06T	A07T	A08T	A09T	Mean
Sensor	61.0	45.8	68.2	39.4	38.0	37.7	56.3	67.3	73.4	54.1
Source	62.4	51.3	70.9	46.3	47.6	43.7	67.2	71.9	74.5	59.7

**Table 2:** 10 × 10-fold cross-validation performance in terms of mean Cohen's kappa value, of the developed method in source and sensor level, and the method developed by the winner of BCI Competition IV, dataset 2a.

Subject	A01T	A02T	A03T	A04T	A05T	A06T	A07T	A08T	A09T	Mean
Sensor	0.48	0.27	0.57	0.19	0.17	0.17	0.42	0.56	0.64	0.39
Source	0.50	0.34	0.61	0.30	0.30	0.26	0.56	0.63	0.66	0.46
Winner FBCSP	0.76	0.47	0.83	0.48	0.60	0.34	0.86	0.80	0.78	0.65

**Table 3:** Sensor method classification sensitivity (true positive rate).

Sensitivity	Left (%)	Right (%)	Foot (%)	Tongue (%)
A01T	49.03	70.00	47.92	75.56
A02T	38.75	41.11	61.81	42.22
A03T	80.14	80.83	50.14	60.83
A04T	34.86	37.64	37.92	46.53
A05T	43.33	48.33	25.83	32.08
A06T	35.42	38.33	50.56	27.64
A07T	68.61	57.08	44.03	56.25
A08T	73.47	61.67	65.28	69.31
A09T	81.67	68.89	67.64	78.47
Mean	56.14	55.99	50.12	54.32

**Table 4:** Sensor method classification specificity (true negative rate).

Specificity	Left (%)	Right (%)	Foot (%)	Tongue (%)
A01T	85.28	84.86	88.33	89.03
A02T	79.63	80.83	87.64	79.86
A03T	91.67	93.47	86.76	85.42
A04T	78.94	77.31	82.59	80.14
A05T	79.40	80.83	79.03	77.27
A06T	80.93	77.96	79.91	78.52
A07T	87.04	85.14	82.50	87.31
A08T	94.21	87.55	83.98	90.83
A09T	93.47	92.78	86.67	92.64
Mean	85.62	84.53	84.16	84.56

**Table 5:** Source method classification sensitivity (true positive rate).

Sensitivity	Left (%)	Right (%)	Foot (%)	Tongue (%)
A01T	55.97	70.56	52.64	70.69
A02T	49.72	38.33	67.08	50.14
A03T	81.25	81.39	57.92	63.06
A04T	49.44	38.61	48.47	46.53
A05T	71.39	59.17	25.00	37.92
A06T	47.36	44.58	59.03	24.44
A07T	88.47	72.50	45.56	60.56
A08T	77.22	75.14	60.14	76.11
A09T	84.58	70.28	65.00	76.94
Mean	67.27	61.17	53.43	56.27

**Table 6:** Source method classification specificity (true negative rate).

Specificity	Left (%)	Right (%)	Foot (%)	Tongue (%)
A01T	86.20	85.88	88.80	89.07
A02T	76.90	84.07	88.33	85.79
A03T	89.63	93.29	89.63	88.66
A04T	75.46	84.07	84.40	83.75
A05T	79.12	80.37	86.76	84.91
A06T	78.15	78.75	82.08	86.16
A07T	88.15	89.17	87.69	90.69
A08T	93.70	90.51	86.99	91.67
A09T	92.27	91.85	88.52	92.96
Mean	84.40	86.44	87.02	88.18

## Data Availability

The BCI Competition IV dataset is available at http://www.bbci.de/competition/iv/. The data from the hereby described analysis can be made available from the authors upon request.

## References

[1] Hochberg L. R., Bacher D., Jarosiewicz B. (2012). Reach and grasp by people with tetraplegia using a neurally controlled robotic arm. *Nature*.

[2] Lee B., Liu C. Y., Apuzzo M. L. J. (2013). A primer on brain–machine interfaces, concepts, and technology: a key element in the future of functional neurorestoration. *World Neurosurgery*.

[3] Nicolas-Alonso L. F., Gomez-Gil J. (2012). Brain computer interfaces, a review. *Sensors*.

[4] Harris J., Hebert A. (2015). Utilization of motor imagery in upper limb rehabilitation: a systematic scoping review. *Clinical Rehabilitation*.

[5] Kraeutner S., Gionfriddo A., Bardouille T., Boe S. (2014). Motor imagery-based brain activity parallels that of motor execution: evidence from magnetic source imaging of cortical oscillations. *Brain Research*.

[6] Shih J. J., Krusienski D. J., Wolpaw J. R. (2012). Brain-computer interfaces in medicine. *Mayo Clinic Proceedings*.

[7] Saimpont A., Lafleur M. F., Malouin F., Richards C. L., Doyon J., Jackson P. L. (2013). The comparison between motor imagery and verbal rehearsal on the learning of sequential movements. *Frontiers in Human Neuroscience*.

[8] Meng J., Zhang S., Bekyo A., Olsoe J., Baxter B., He B. (2016). Noninvasive electroencephalogram based control of a robotic arm for reach and grasp tasks. *Scientific Reports*.

[9] Galán F., Nuttin M., Lew E. (2008). A brain-actuated wheelchair: asynchronous and non-invasive Brain–computer interfaces for continuous control of robots. *Clinical Neurophysiology*.

[10] He B., Baxter B., Edelman B. J., Cline C. C., Ye W. W. (2015). Noninvasive brain-computer interfaces based on sensorimotor rhythms. *Proceedings of the IEEE*.

[11] Edelman B. J., Baxter B., He B. (2016). EEG source imaging enhances the decoding of complex right-hand motor imagery tasks. *IEEE Transactions on Biomedical Engineering*.

[12] Nunez P. L., Srinivasan R. (2006). *Electric Fields in the Brain: The Neurophysics of EEG*.

[13] Michel C. M., Murray M. M., Lantz G., Gonzalez S., Spinelli L., Grave de Peralta R. (2004). EEG source imaging. *Clinical Neurophysiology*.

[14] He B., Yang L., Wilke C., Yuan H. (2011). Electrophysiological imaging of brain activity and connectivity-challenges and opportunities. *IEEE Transactions on Biomedical Engineering*.

[15] Ramoser H., Müller-Gerking J., Pfurtscheller G., Muller-Gerking J., Pfurtscheller G. (2000). Optimal spatial filtering of single trial EEG during imagined hand movement. *IEEE Transactions on Rehabilitation Engineering*.

[16] Bashashati A., Fatourechi M., Ward R. K., Birch G. E. (2007). A survey of signal processing algorithms in brain-computer interfaces based on electrical brain signals. *Journal of Neural Engineering*.

[17] Lemm S., Blankertz B., Curio G., Müller K.-R. (2005). Spatio-spectral filters for improving the classification of single trial EEG. *IEEE Transactions on Biomedical Engineering*.

[18] Camilleri K. P., Falzon O., Camilleri T., Fabri S. G., Sakkalis V. (2014). Phase variants of the common spatial patterns method. *Modern Electroencephalographic Assessment Techniques, Neuromethods*.

[19] Brandon T. H., Tiffany S. T., Obremski K. M., Baker T. B. (1990). Postcessation cigarette use: the process of relapse. *Addictive Behaviors*.

[20] Lotte F., Guan C. (2011). Regularizing common spatial patterns to improve BCI designs: unified theory and new algorithms. *IEEE Transactions on Biomedical Engineering*.

[21] Ang K. K., Chin Z. Y., Wang C., Guan C., Zhang H. (2012). Filter bank common spatial pattern algorithm on BCI competition IV datasets 2a and 2b. *Frontiers in Neuroscience*.

[22] Bai X., Wang X., Zheng S., Yu M. The offline feature extraction of four-class motor imagery EEG based on ICA and Wavelet-CSP.

[23] Tangermann M., Müller K.-R., Aertsen A. (2012). Review of the BCI Competition IV. *Frontiers in Neuroscience*.

[24] Pfurtscheller G., Brunner C., Schlögl A., Lopes da Silva F. H. (2006). Mu rhythm (de)synchronization and EEG single-trial classification of different motor imagery tasks. *Neuroimage*.

[25] Oostenveld R., Fries P., Maris E., Schoffelen J.-M. (2011). FieldTrip: open source software for advanced analysis of MEG, EEG, and invasive electrophysiological data. *Computational Intelligence and Neuroscience*.

[26] Buzsáki G., Anastassiou C. A., Koch C. (2012). The origin of extracellular fields and currents—EEG, ECoG, LFP and spikes. *Nature Reviews Neuroscience*.

[27] Hallez H., Vanrumste B., Grech R. (2007). Review on solving the forward problem in EEG source analysis. *Journal of NeuroEngineering and Rehabilitation*.

[28] Holmes C. J., Hoge R., Collins L., Woods R., Toga A. W., Evans A. C. (1998). Enhancement of MR images using registration for signal averaging. *Journal of Computer Assisted Tomography*.

[29] Gramfort A., Papadopoulo T., Olivi E., Clerc M. (2010). OpenMEEG: opensource software for quasistatic bioelectromagnetics. *BioMedical Engineering Online*.

[30] Fang Q., Boas D. A. Tetrahedral mesh generation from volumetric binary and grayscale images.

[31] Geddes L. A., Baker L. E. (1967). The specific resistance of biological material–a compendium of data for the biomedical engineer and physiologist. *Medical & Biological Engineering*.

[32] Grech R., Cassar T., Muscat J. (2008). Review on solving the inverse problem in EEG source analysis. *Journal of NeuroEngineering and Rehabilitation*.

[33] Hämäläinen M. S., Ilmoniemi R. J. (1994). Interpreting magnetic fields of the brain: minimum norm estimates. *Medical & Biological Engineering & Computing*.

[34] Tadel F., Baillet S., Mosher J. C., Pantazis D., Leahy R. M. (2011). Brainstorm: a user-friendly application for MEG/EEG Analysis. *Computational Intelligence and Neuroscience*.

[35] Cincotti F., Mattia D., Aloise F. (2008). High-resolution EEG techniques for brain–computer interface applications. *Journal of Neuroscience Methods*.

[36] Athanasiou A., Xygonakis I., Pandria N. (2017). Towards rehabilitation robotics: off-the-shelf BCI control of anthropomorphic robotic arms. *Biomed Research International*.

[37] Dornhege G., Blankertz B., Curio G., Muller K.-R. (2004). Boosting bit rates in noninvasive EEG single-trial classifications by feature combination and multiclass paradigms. *IEEE Transactions on Biomedical Engineering*.

[38] Rokach L. (2010). Ensemble-based classifiers. *Artificial Intelligence Review*.

[39] Park S.-H., Lee D., Lee S.-G. (2018). Filter bank regularized common spatial pattern ensemble for small sample motor imagery classification. *IEEE Transactions on Neural Systems and Rehabilitation Engineering*.

[40] Chuang C.-H., Ko L.-W., Lin Y.-P., Jung T.-P., Lin C.-T. (2014). Independent component ensemble of EEG for brain–computer interface. *IEEE Transactions on Neural Systems and Rehabilitation Engineering*.

[41] Ang K. K., Chin Z. Y., Zhang H., Guan C. Filter Bank Common Spatial Pattern (FBCSP) in brain-computer interface.

[42] Cohen J. (1960). A coefficient of agreement for nominal scales. *Educational and Psychological Measurement*.

[43] Landis J. R., Koch G. G. (1977). The measurement of observer agreement for categorical data. *Biometrics*.

[44] Ryu S. I., V Shenoy K. (2009). Human cortical prostheses: lost in translation?. *Neurosurgical Focus Other Titles*.

[45] Wolpaw J. R., Birbaumer N., McFarland D. J., Pfurtscheller G., Vaughan T. M. (2002). Brain–computer interfaces for communication and control. *Clinical Neurophysiology*.

[46] He B., Ding L. (2013). Electrophysiological mapping and neuroimaging. *Neural Engineering*.

[47] Kamousi B., Amini A. N., He B. (2007). Classification of motor imagery by means of cortical current density estimation and Von Neumann entropy. *Journal of Neural Engineering*.

[48] Athanasiou A., Klados M. A., Styliadis C., Foroglou N., Polyzoidis K., Bamidis P. D. (2016). Investigating the role of alpha and beta rhythms in functional motor networks. *Neuroscience*.

[49] Mattia D., Cincotti F., Astolfi L. (2009). Motor cortical responsiveness to attempted movements in tetraplegia: evidence from neuroelectrical imaging. *Clinical Neurophysiology*.

[50] Akalin Acar Z., Makeig S. (2013). Effects of forward model errors on eeg source localization. *Brain Topography*.

[51] Ramon C., Schimpf P., Haueisen J., Holmes M., Ishimaru A. (2003). Role of soft bone, CSF and gray matter in EEG simulations. *Brain Topography*.

[52] Vorwerk J., Cho J.-H., Rampp S., Hamer H., Knösche T. R., Wolters C. H. (2014). A guideline for head volume conductor modeling in EEG and MEG. *Neuroimage*.

[53] Vorwerk J., Oostenveld R., Piastra M. C., Magyari L., Wolters C. H. (2018). The FieldTrip-SimBio pipeline for EEG forward solutions. *BioMedical Engineering Online*.

[54] Wronkiewicz M., Larson E., Lee A. K. C. (2015). Leveraging anatomical information to improve transfer learning in brain-computer interfaces. *Journal of Neural Engineering*.

[55] Athanasiou A., Arfaras G., Pandria N. (2017). Wireless brain-robot interface: user perception and performance assessment of spinal cord injury patients. *Wireless Communications and Mobile Computing*.

